# Speciation Distribution of Heavy Metals in Uranium Mining Impacted Soils and Impact on Bacterial Community Revealed by High-Throughput Sequencing

**DOI:** 10.3389/fmicb.2019.01867

**Published:** 2019-08-13

**Authors:** Shiqi Xiao, Qian Zhang, Xiaoming Chen, Faqin Dong, Hao Chen, Mingxue Liu, Imran Ali

**Affiliations:** ^1^School of Life Sciences and Engineering, Southwest University of Science and Technology, Mianyang, China; ^2^National Co-Innovation Center for Nuclear Waste Disposal and Environmental Safety, Southwest University of Science and Technology, Mianyang, China; ^3^State Key Laboratory of NBC Protection for Civilian, Beijing, China; ^4^Sichuan Institute of Atomic Energy, Chengdu, China; ^5^Institute of Biochemistry, University of Balochistan, Quetta, Pakistan

**Keywords:** uranium mine, heavy metal pollution, chromium, bacterial community, high-throughput sequencing

## Abstract

This study investigated the influence of heavy metals on bacterial community structure in a uranium mine. Soils from three differently polluted ditches (Yangchang ditch, Zhongchang ditch, and Sulimutang ditche) were collected from Zoige County, Sichuan province, China. Soil physicochemical properties and heavy metal concentrations were measured. Differences between bacterial communities were investigated using the high-throughput sequencing of the 16S rRNA genes. The obtained results demonstrated that bacterial richness index (Chao and Ace) were similar among three ditches, while the highest bacterial diversity index was detected in the severely contaminated soils. The compositions of bacterial communities varied among three examined sites, but Proteobacteria and Acidobacteria were abundant in all samples. Redundancy analysis revealed that soil organic matter, Cr and pH were the three major factors altering the bacterial community structure. Pearson correlation analysis indicated that the most significant correlations were observed between the contents of non-residual Cr and the abundances of bacterial genera, including *Thiobacillus, Nitrospira*, and other 10 genera. Among them, the abundances of *Sphingomonas* and *Pseudomonas* were significant and positively correlated with the concentrations of non-residual U and As. The results highlighted the factors influencing the bacterial community in uranium mines and contributed a better understanding of the effects of heavy metals on bacterial community structure by considering the fraction of heavy metals.

## Introduction

With the rapid development of nuclear technology, uranium mining, and smelting activities have increased. Activities associated with mining have generated enormous waste materials containing uranium and other toxic metals (Ilton et al., 2008). Uranium is considered one of the most hazardous pollutants among metals, due to its chemical and radiological toxicities (Qi et al., 2019). Uranium occurs environmentally in two oxidation states: the oxidized form U (VI) and the reduced form U (IV). The strong oxidizing power of U (VI) shows a predominance of chemical toxicity. Oxidative DNA damage causes long-term genotoxic effects in the form of mutagenesis, carcinogenesis, and other pathologies (Gudkov et al., 2016). It is of great importance to study the influence of uranium pollution on soil ecological environment.

Microorganisms are always considered the sensitive indicators of environmental stress (Giller et al., 2009; Bano et al., 2018). In the soil ecosystem, microorganisms play important roles in energy flow, nutrient cycling, and organic matter recycling (Chodak et al., 2013). They may act as a nutrient source in soils and participate in the humification processes, degradation of pollutants, and maintenance of soil structure (Verstraete and Top, 1999; Preston et al., 2001). A well-functioning microbial community is a precondition for soil fertility. Research on microbial communities in uranium mines will not only help in understanding the habituating features of microbes in heavy metals and radioactive environments but will also elaborate the possibilities of bioremediation of uranium contaminated soil (Chen et al., 2012). Mondani et al. (2011) studied the effect of uranium on bacterial communities and the results indicated that sequences related to iron-reducing bacteria and iron-oxidizing species are specific to uraniferous samples. Kenarova et al. (2014) found that the physiological diversity of bacteria was site specific in three abandoned mines. An investigation of uranium mill tailings revealed that Proteobacteria was the most dominant flora (Yan and Luo, 2015). These reports help us to understand microbial community structures under the stress of a uranium mine. The existing information in literature about the effect of heavy metals and uranium on soil microorganisms is limited. Meanwhile, the total contents of heavy metals may not necessarily be an indicator reflecting adverse effects on microbiomes, because the toxic effects are dependent not only dependent on the intrinsic toxicity and concentration of contaminant but also on geochemical phases in which it is present in soil (Olaniran et al., 2017). The geochemical forms of heavy metals are varied in natural environment, including exchangeable, carbonate, Fe–Mn bound, organic, and residual fractions (Tessier et al., 1979; Neto et al., 2016; Palleiro et al., 2016). These forms are directly related to the interactions process, bioavailability, transfer behaviors and potential toxicity of many metals (Najamuddin et al., 2016). Exchangeable forms can be easily absorbed by microorganisms, while carbonate, Fe–Mn bound and organic fractions can be translated into activated forms as a result of changes in some conditions of soil environment, like pH, salinity, and redox potential (Ma et al., 2016; Huang et al., 2017). In contrast, residual fractions appear to be biologically inactive since they are embedded into the crystalline lattices of the soil clay (Palleiro et al., 2016). Estimations of heavy metal contents in various geochemical fractions are helpful for evaluating the effects of heavy metals on microorganisms.

In recent years, the next-generation sequencing techniques have widely been used for investigating the complexity of microbiomes (Kuppusamy et al., 2016; Li X. et al., 2017; Narendrula-Kotha and Nkongolo, 2017), such as Illumina sequencing of 16S rRNA amplicons can provide much higher resolution approaches studying the phylogenetic composition of microbial communities and obtaining an enormous number of sequences in a short time (Fierer et al., 2012). This method is very useful for exploring the relationships between contents of heavy metals and diversity and abundances of soil microbial communities. In this work we investigated the structure of indigenous bacterial community in uranium mine area using the 16S rRNA gene amplicon sequencing. We collected samples from contaminated fields located in Zoige County in Sichuan Province of China. Soils at these sites were polluted, mostly with uranium and heavy metals, as a result of the exploitation of mining. The main objectives of the study were to characterize the diversity and composition of bacterial communities in uranium mine and to evaluate the relationships between the contents of contaminants and bacterial community structures. This research can provide a deep understanding of microbiomes revealed by high-throughput sequencing in polymetallic and uranium contaminated soils.

## Materials and Methods

### Sampling of Soils Contaminated With Uranium and Heavy Metals

**FIGURE 1:**
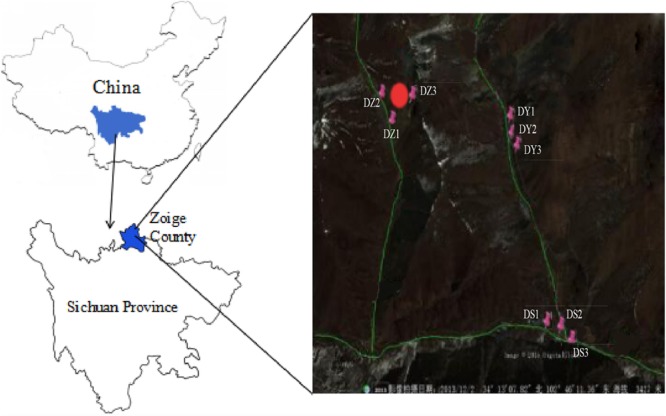
Location of samples in the mine area. “DZ,” soil samples in ditch Z; “DY,” soil samples in ditch Y; “DS,” soil samples in ditch S. The red mark represents the sampling sites. The three green lines represent the three ditches, with DZ on the left, DY on the right, and DS at the bottom. The direction of flow was from top to bottom and left to right (©2019 satellite map).

The significant part of Zoige uranium mine is located in Zoige County in Sichuan province and the rest of mine extends to the east, to Diebu County in Gansu province of China. The ore belt is developed from west to east and is accompanied by heavy metals. The total length of the ore belt is approximately 50 km, with a width of approximately 10 km wide. It consists of mine deposits of different sizes. This mining was explored during 1970–1990s and has been abandoned from approximately 20 years (Yang et al., 2018). Soils of the region have been seriously polluted by numerous metals due to mining activities. The samples were collected from the Zoige mine field (102°45′–102°46′E, 34°12′N–34°13′N) in April 2014 along with the Yangchang (DY1, DY2, and DY3), Zhongchang (DZ1, DZ2, and DZ3), and Sulimutang (DS1, DS2, and DS3) ditches. DZ represents the sample collected from a site close to the center of the mine. DY was a mineral-leaching area. DS was far away region from mine, which were slightly contaminated with heavy metals. The elevation of the mine is approximately 3300–3700 m; DZ and DS have the highest and lowest average elevation, respectively. All the samples were collected and labeled according to their flow direction of flow from upstream to downstream. The sampling points were first mapped (Geographic Information System location), and a total of nine points were selected (Figure 1). Top soil from the 5–20 cm layer was collected. Sampling at the 2 m^2^ area of each site and each sample was comprised of five subsamples. The subsamples were well-mixed after removing large stones and plant residues. The soil samples were sealed in plastic bags, immediately moved to the laboratory in cooling boxes. These samples were separated into two parts. One part was stored in −80°C freezer for molecular analysis. And the other part was stored at 4°C for chemical analysis.

### Total Content and Speciation of Uranium and Heavy Metals in Soils

Soil moisture content was dried at 105°C for 24 h. Soil pH was determined using a calibrated pH meter (soil/solution ratio of 1:2.5). Soil organic matter (SOM) was tested by the K_2_Cr_2_O_7_ colorimetric method. The five-step sequential extraction method was conducted to determine the speciations of heavy metals in soil samples (Tessier et al., 1979). Five fractions were determined including exchangeable, carbonate, Fe–Mn bound, organic and residual fractions. Details of extraction process can be found in several references (Tessier et al., 1979; Buccolieri et al., 2004; Xiao et al., 2015). The contents of heavy metals in extracted solution were analyzed by the inductively coupled plasma atomic emission spectrometry (ICP-AES, ICAP6500, Thermo Scientific). Total contents of heavy metals were also determined by ICP-AES after soil samples were digested in the HClO_4_-HNO_3_-HF mixture in Teflon tubes.

Heavy metals are the natural component of soil. It is very important to distinguish between the natural background values and anthropogenic inputs. To evaluate the soil contamination rate, the geo-accumulation index *I*_geo_) can be applied. This index was used to assess the presence and intensity of anthropogenic contamination deposition on soil. It can be calculated according to the following formula:

$$\begin{array}{c} I_{\mathrm{geo}}=\;\log_{2}\frac{Cn}{1.5\times Bn} \end{array}$$

C_n_ is the measured content of metal n, and B_n_ is the geological background value of metal n. Factor 1.5 is the background matrix correlation factor due to lithospheric effects. The background value for As, Cr, Mo, Sr, V, Zn, and U is 11, 61, 2.0, 166, 82, 74, and 3.0 mg kg^–1^, respectively (Chen et al., 1991). The *I*_geo_ of each heavy metal can be classified as: unpolluted (*I*_geo_ ≤ 0); weakly polluted (0 < *I*_geo_ ≤ 1); moderately polluted (1 < *I*_geo_ ≤ 2); moderately to heavily polluted (2 < *I*_geo_ ≤ 3); heavily polluted (3 < *I*_geo_ ≤ 4); heavily to extremely polluted (4 < *I*_geo_ ≤ 5); extremely polluted (*I*_geo_ > 5) (Muller, 1981).

### DNA Extraction, PCR Amplification, and Sequencing

The total microbial DNA was extracted from 0.5 g soil using a PowerSoil^®^ DNA Isolation kit (Omega Bio-tek, Inc., Doraville, GA, United States) according to the manufacturer’s instructions. The extracted DNA was analyzed by 1% agar gel electrophoresis and used as a template for amplification. The average concentration of DNA in nine soil samples was 74.2 ng/l. The universal bacterial primer 27F (5′-AGAGTTTGATCCTGGCTCAG-3′) and 338R (5′-TGCTG CCTCCCGTAGGAGT-3′) (Ravel et al., 2011) were used to amplify the hypervariable fragments (V1–V3) of the 16S rRNA. PCR amplification was performed using TransGen AP221-02:TransStart FastPfu DNA Polymerase (TransGen Biotech, Beijing, China) on an ABI GeneAmp^®^ 9700 system, in triplicate assays. The PCR system included 4 μl 5× Buffer, 2 μl 2.5 mM dNTPs, 5 μM of each forward and reverse primer, 0.2 μl rTaq Polymerase 0.2 μl Bovine Serum Albumin, 10 ng of template DNA was added in ddH_2_O tp 20 μl. PCR procedure was set as: an initial denaturing step at 95°C for 3 min, followed by 35 cycles at 95°C (30 s), 55°C (30 s), 72°C (45 s) and a final extension step at 72°C for 10 min. Amplicons were extracted from 2% agarose gels and purified with an AxyPrep DNA Gel Extraction Kit (Axygen Biosciences, Union City, CA, United States) according to the manufacturer instructions and quantified using QuantiFluor^TM^ -ST (Promega, United States). The sequencing library was constructed using mixture of 150 ng of each recovered PCR product. Sequencing were conducted on an Illumina Miseq platform by the Majorbio Bio-Pharm Technology, Co., Ltd. (Shanghai, China). The raw reads had been submitted to the NCBI and the accession numbers of sequences for DY1, DY2, DY3, DZ1, DZ2, DZ3, DS1, DS2, and DS3 was SRR9611297, SRR9611293, SRR9611298, SRR9611292, SRR9611295, SRR9603206, SRR9611294, SRR9611299, and SRR9611296.

### Data Processing and Statistical Analyses

The raw data (FASTQ files) were quality-filtered using QIIME (version 1.9.1) (Caporaso et al., 2010) based on the following criteria: (a) 300 bp reads were truncated at any site with an average quality score < 20 over a 50 bp sliding window, and the truncated reads below 50 bp were discarded; (b) the exact barcodes were matched, two-nucleotide mismatch in primer matching and reads containing ambiguous base calls were removed and (c) only sequences with overlaps longer than 10 bp were merged together. Reads that could not be merged were discarded.

The filtered sequences were clustered into operational taxonomic units (OTUs) at 97% sequence similarity cut-off using Usearch (Version 7.1) (Edgar, 2010), and representative sequences were selected after the removal of putative chimeras. The most abundant sequence within each OTU was selected to represent the respective OTU in subsequent assays. The taxonomic identity of each phylotype was determined using RDP Classifier (Cole et al., 2009) with a 70% bootstrap score; the database selected was Silva (Release 128). Additionally, the richness indices Chao and ACE were evaluated with Mothur software (version 1.30.1) (Kozich et al., 2013). The Shannon and Simpson diversity index were also measured with alpha diversity analyses.

Detrended correspondence analysis (DCA) was used to identify the models. The results showed a linear model according to the length of the first axis (1.729). Based on the results of DCA, RDA was carried to determine which environmental factors exert significant impact on bacterial community structure, which were performed using Canoco software (version 4.5, for Windows). Hierarchical clustering analysis based on Bray-Curtis dissimilarity (Bray and Curtis, 1957) was performed by QIIME. One-way ANOVA analyses and Tukey test were applied to identify the significant differences in soil heavy metal content and diversity index among soil samples. Pearson correlation analysis was conducted to investigate correlation between heavy metals content and species richness (genus level) using SPSS version 17.0.

## Results

### Content of Uranium and Heavy Metals in Soil

**FIGURE 2:**
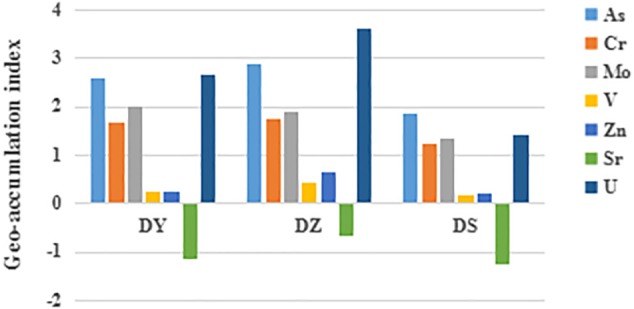
Geo-accumulation index (*I*_geo_) of uranium and heavy metals in soil samples from different ditches. DY: *I*_geo_ of DY; DZ: *I*_geo_ of DZ; DS: *I*_geo_ of DS.

Soil pH, moisture content (MC), SOM and concentration of heavy metals are shown in Table 1. The contents of SOM and MC in DS were significantly higher than those in DY and DZ. Soil pH was the highest in DS, but no significant difference was observed among three sites. *I*_geo_ was taken into consideration to assess the pollution level of heavy metals in this region, which takes the geochemical background value as a reference. Figure 2 shows that the *I*_geo_ value for Cr and Mo in all soil samples of DY, DZ, and DS were classified into moderately polluted category. Comparatively, V and Zn in all soils showed “weakly polluted” level according to the *I*_geo_ values. All negative *I*_geo_ values for Sr were obtained in soils, indicating an “unpolluted” level. For As and U, the pollution level was varied in different ditch. In DS, soil samples were moderately polluted by these two kinds of heavy metals. However, *I*_geo_ of As and U was higher than 2.0 in DY, indicating that soils in this ditch was moderately to heavily polluted by As and U. Considerably, DZ was heavily polluted by U according to *I*_geo_ value. Generally, the pollution levels in DY and DZ were relatively more serious than DS. The pollution level of determined metals followed the sequence As ≈ U > Mo > Cr > Zn > V > Sr.

**TABLE 1:** Mean values of uranium and heavy metals contents in three soil samples collected from the same ditch (mean ± SE).

	**As**	**Cr**	**Mo**	**Sr**	**V**	**Zn**	**U**	**SOM**	**MC**	
**Ditches**	**(mg kg^–1^)**	**(mg kg^–1^)**	**(mg kg^–1^)**	**(mg kg^–1^)**	**(mg kg^–1^)**	**(mg kg^–1^)**	**(mg kg^–1^)**	**(g kg^–1^)**	**(%)**	**pH**
DY	98.4 ± 10.55a	290.2 ± 31.66a	11.99 ± 3.144a	113.6 ± 4.051a	147.8 ± 21.08a	131.8 ± 4.481*ab*	28.31 ± 14.14*ab*	21.01 ± 1.813b	17.24 ± 1.174b	7.183 ± 0.221a
DZ	123.1 ± 6.41a	304.9 ± 55.80a	11.07 ± 4.126a	155.0 ± 46.61a	165.8 ± 21.83a	174.9 ± 10.41a	54.99 ± 26.23a	20.91 ± 3.80b	14.51 ± 1.342b	7.501 ± 0.261a
DS	59.86 ± 16.08b	217.7 ± 11.58a	7.547 ± 0.887a	104.1 ± 11.69a	139.7 ± 2.838a	127.4 ± 30.29b	11.92 ± 3.359b	31.33 ± 2.824a	24.51 ± 1.385a	7.713 ± 0.340a

*Different letters in the same column indicate that the differences between soil samples are significant at *p* < 0.05.*

**FIGURE 3:**
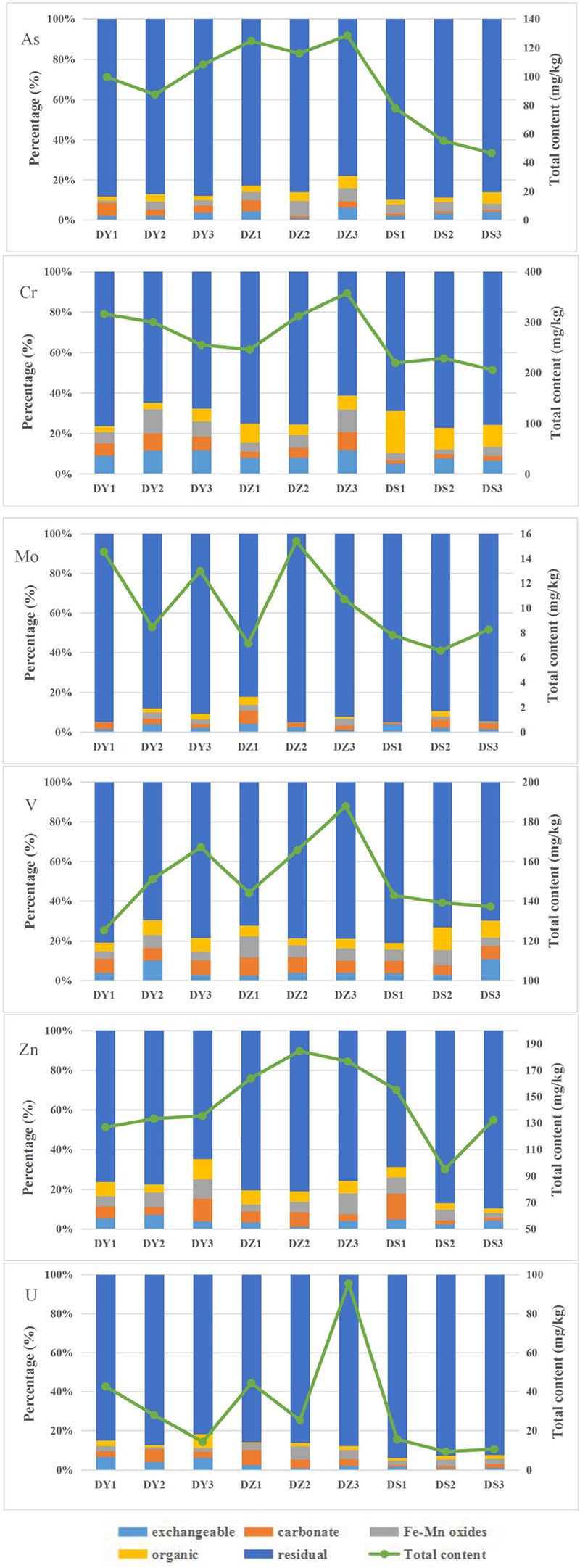
Uranium and heavy metals total contents and their distributions in different geochemical phases of the nine soil samples from the Zoige uranium mine, China.

A well-established sequential extraction method (Tessier et al., 1979) was performed to investigate the fractional distribution of heavy metals in soil samples. The values of heavy metals pertaining to various geochemical phases of each site are shown in Figure 3 On average, all the studied heavy metals showed dominant levels in residual fractions, with mean value of 86.06, 71.37, 91.3, 75.93, 77.89, and 87.99% of the mean total concentration for As, Cr, Mo, V, Zn, and U, respectively. For Mo, V and U, the carbonate fraction was the second abundant form, accounting for 2.96, 6.72, and 3.64% of the mean total concentration, respectively. The mean proportions of Mo, V, and U in the exchangeable fraction were 2.59, 4.97, and 2.88%, while 1.67, 6.22, and 3.14% in the Fe–Mn bound fraction and 1.48, 6.16, and 2.34% in the organic fraction. The exchangeable was the most abundant form of non-residual Cr (8.9%). For As and Zn, Fe–Mn bound fraction was the most predominant form of the non-residual fraction.

### Bacterial Community Structure

A total of 79363 high-quality 16S rRNA gene reads were obtained for all nine samples, with an average of 8818 (ranging from 5591 to 12111) reads per community. In total, 8384 OTUs were identified. The rarefaction curve (Supplementary Figure S1) reached saturation, indicating the sequencing depths were reasonable for further analysis. Bacterial alpha diversity was further estimated based on 97% OTUs. As shown in Table 2, the OTU number showed slight change in three areas. The Chao1 estimator and ACE were used to estimate the amounts of OTU, considering the number of singletons per sample, since they may suggest additional undetected phylotypes (Chao, 1984). Both parameters further confirmed the order of bacterial richness (OTU) and were similar among three ditches. However, alpha diversity indexes, including Simpson and Shannon diversity index were found in significant differences between DS and the other two sites. The highest diversity was found in DY, with indices ranging from 6.09 to 6.15, while the diversity index in DS was the lowest, with indices ranging from 5.78 to 5.84.

**TABLE 2:** Bacterial diversity indices and estimated bacterial OTUs of soil samples collected from three ditches.

**Ditches**	**OTU**	**Ace**	**Chao**	**Shannon**	**Simpson**
DY	911 ± 119a	1235 ± 47.6a	1202 ± 50.2a	6.12 ± 0.03a	0.0045 ± 0.0003c
DZ	1060 ± 131a	1293 ± 68.6a	1257 ± 34.9a	6.057 ± 0.036a	0.0058 ± 0.0004b
DS	823 ± 107a	1192 ± 33.1a	1178 ± 47.17a	5.81 ± 0.03b	0.0074 ± 0.0004a

*Different letters in the same column indicate that the differences between soil samples are significant at *p* < 0.05.*

**FIGURE 4:**
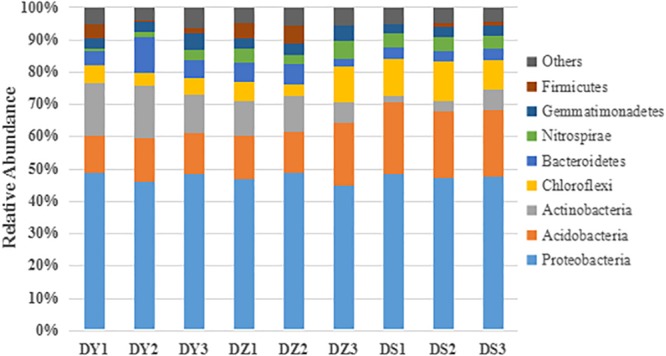
Taxonomic composition of the dominant bacteria at the phylum level in different soil samples. Others represent the relative abundance of the phyla outside the eight phyla.

Taxonomic information at the phylum level is shown in Figure 4. Among all identified bacterial phylum in the studied regions, Proteobacteria and Acidobacteria were two major phyla. The remaining dominant community was composed of Actinobacteria, Bacteroidetes, Chloroflexi, Firmicutes, Gemmatimonadetes, and Nitrospirae. These eight phyla totally accounted for a high proportion (93.54–96.2%) in soil bacterial communities. The mean percentage of Proteobacteria in soils were much higher than other phyla, which also had the similar distribution pattern in three ditches (47.75% in DY, 46.89% in DZ, and 47.84% in DS). The site-specific trend influenced bacterial distribution. Gemmatimonadetes (3.90% in DY, 3.69% in DZ, and 3.06% in DS) and Bacteroidetes (6.93% in DY, 4.77% in DZ, and 3.52% in DS) were more abundant in DY, but Nitrospirae (1.79% in DY, 4.32% in DZ, and 4.01% in DS) and Firmicutes (2.14% in DY, 3.57% in DZ, and 0.83% in DS) were more abundant in DZ. The highest proportion of Chloroflexi (4.84% in DY, 7.01% in DZ, and 10.83% in DS) was in DS. In addition, the abundance of Phyla Actinobacteria (14.98% in DY, 9.24% in DZ, and 3.88% in DS) was significantly higher in DY than in DS, while Acidobacteria (12.50% in DY, 15.23% in DZ, and 21.11% in DS) was significantly lower in DY and DZ than DS.

**FIGURE 5:**
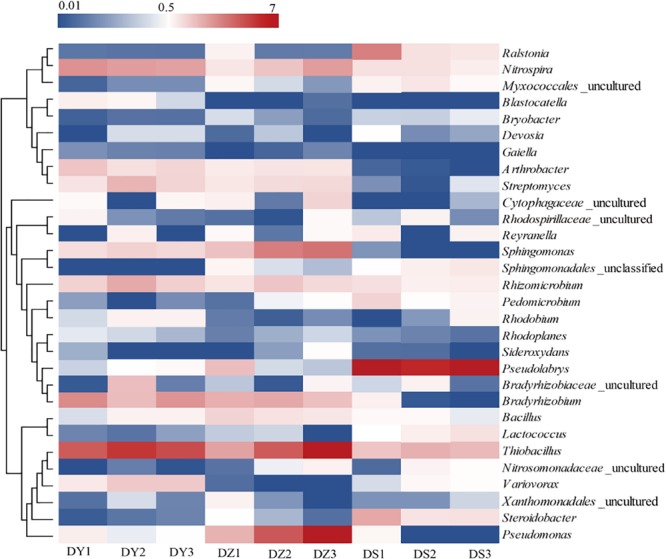
Heatmap analysis of the dominant genera in different soil samples. Changes in bacterial community compositions was depicted by the color intensity ranged from 0.01 to 7 (blue to red).

At the genus level, most of genera showed different abundance in different ditch (Figure 5). *Thiobacillus, Nitrospira*, and *Sphingomonas* were dominant in all soil samples, in which these bacteria accounted for 10.06, 8.28, and 4.37% of the total community in DY, DZ, and DS respectively. In DZ, the specifically dominant members were *Bacillus* and *Pseudomonas*, while DY was specifically dominated by *Variovorax*. Moreover, *Arthrobacter* (1.59% DY, 1.13% DZ, and 0.12% DS), *Streptomyces* (1.7% DY, 1.3% DZ, and 0.27% DS), *Bradyrhizobium* (2.89% DY, 2.37% DZ, and 0.34% DS) and *Rhizomicrobium* (1.97% DY, 1.51% DZ, and 0.43% DS) were more abundant in DY and DZ than those in DS. However, *Ralstonia* and *Psedulabrys* were predominated in DS, totally accounting for 13.41% of the total community.

### Relationship Between Bacterial Community Structures and Concentrations of Uranium and Heavy Metals

**FIGURE 6:**
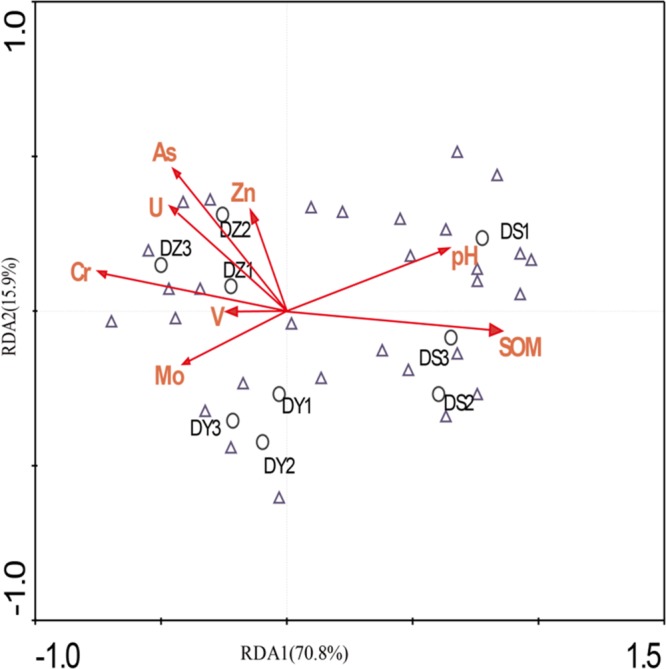
Redundancy analysis of bacterial data and environmental parameters. Samples and species are represented as circles and triangles, respectively. Arrows indicate the directions and magnitudes of heavy metals associated with bacterial community structures.

**FIGURE 7:**
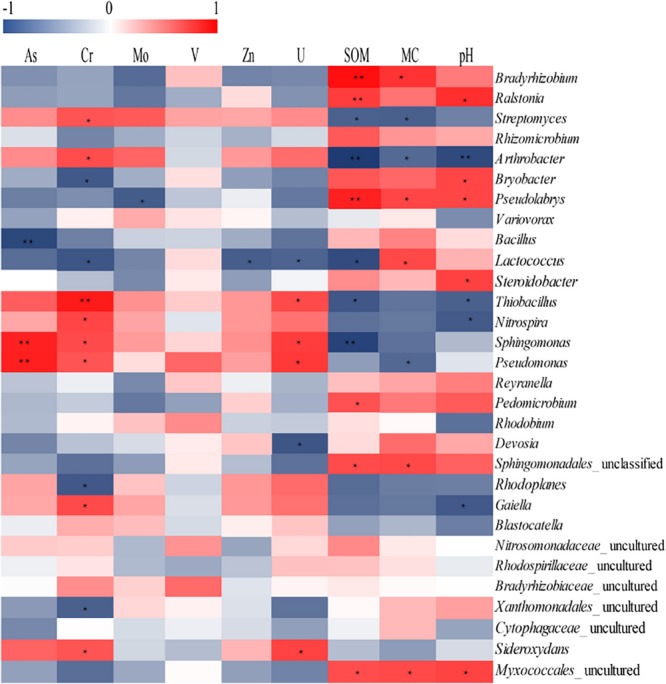
Analysis of correlation between environmental parameters and dominant genera richness. The corresponding values of the heat map is the Pearson correlation coefficient. The value of “r” is between –1 and 1, *r* < 0 is a negative correlation, *r* > 0 is a positive correlation, values marked ^*^ indicate a significance test at *p* < 0.05, and values marked ^∗∗^ indicate a significance test at *p* < 0.01.

Using RDA we revealed a clear relation between bacterial community structure and environmental factors (Figure 6). The length of the arrow representing each parameter is directly proportional to the influence of environmental factors on bacterial community structure. The first two axes of the RDA explained 70.8 and 15.9% of the total variation, respectively. Furthermore, the first dimension (RDA1) was driven by SOM, Cr and pH, while the RDA2 was driven by As and U. The effect of SOM, Cr, and pH on the bacterial communities were significant (*p* < 0.05), indicating that three parameters were the principle factors influencing the bacterial communities. However, the other factors had no obvious effect on bacterial communities. The Pearson correlation was performed to analyze correlations between environmental parameters and bacterial abundance (Figure 7). The abundances of *Thiobacillus, Nitrospira, Sphingomonas, Pseudomonas, Streptomyces, Arthrobacter, Gaiella*, and *Sideroxydans* were significant and positively correlated with the concentrations of non-residual Cr, while the abundances of *Bryobacter, Lactococcus*, and *Rhodoplanes* negatively correlated with concentrations of the same metals. The abundances of *Sphingomonas* and *Pseudomonas* were significant and positively correlated with the concentrations of non-residual U and As. The abundance of *Bradyrhizobium, Lactococcus, Sphingomonadales*_unclassified and *Myxococcales*_uncultured were positively correlated with SOM and MC, while *Lactococcus* was negatively correlated with non-residual Cr, U, and Zn. In addition, *Ralstonia, Bryobacter, Pseudolabrys*, and *Steroidobacter* were positively correlated with pH, while *Thiobacillus, Nitrospira, Arthrobacter*, and *Gaiella* were negatively correlated with it.

## Discussion

### Uranium and Heavy Metals Distribution and Speciation in All Soil Samples

The SOM and MC among three sites decreased as follows: DS > DY > DZ, while the content of heavy metals showed an opposite trend. This may be because the DZ site was close to the center of the polluted area than DS site. The DY site was situated in a mineral-leaching area, where numerous heavy metals could get into soils. Heavy metal contamination in this area originated mainly from the mining activities. The industrial activities were able to constrain the accumulation of organic matter in soil. The MC was highest in DS, which might attribute to the highest content of SOM in DS which could increase soil porosity, thus improving its ability to retain water (Wu et al., 2013).

The value of *I*_geo_ indicated that all three ditches were most seriously polluted by U and As, followed by Mo, Cr, Zn, and V. Sr in all cases did not exceed the background levels. However, *I*_geo_ was calculated based on the total heavy metal concentration, although the toxicity of heavy metals to soil microorganisms are directly dependent from the presence of specific chemical forms in which metals are present in soil (Kot and Namiesñik, 2000; Wang et al., 2007b; Ke et al., 2017). The exchangeable and carbonate fractions can be easily leached in soil and exist in a free form, which allows them to be directly absorbed by microorganisms. An increase of oxidizing conditions in soil can dissolve Fe–Mn bound and the organic matter fractions, transforming these fractions into active forms (Arenas-Lago et al., 2016). Metals of the residual fraction are embedded into the crystalline lattices of the soil clay, which mainly originated from geogenic sources and appear to be rather inactive (Islam et al., 2015). Studies of metal extraction from solution by aquatic organisms verified the high availability of free metal ions (Luoma, 1983). Based on the previous researches, the metals which exist in non-residual forms, are more likely utilized by organisms and may relate to their concentrations in microorganisms (Roosa et al., 2016; Rosado et al., 2016; Yıldırım and Tokalıoğlu, 2016). Consequently, the potential bioavailability of heavy metals is mainly determined by proportions in non-residual fraction.

The distribution of five forms of heavy metals in DY, DZ, and DS were varied. In DY, the exchangeable fraction showed the biggest proportion (6.27% in DY1, 8.9% in DY2, and 6.58% in DY3) among non-residual fractions, which was significantly higher than those of the organic form (3.77% in DY1, 4.23% in DY2, and 4.41% in DY3). Inversely, the organic fraction was the major component of the non-residual fraction in DS, which accounted for 9.76% in DS1, 8.44% in DS2, and 7.46% in DS3, respectively. Compared with the other two ditches, Fe–Mn bound was the most abundant forms of the non-residual fraction in DZ. Generally, the percentage of the non-residual fractions in each ditch followed the order: DY > DZ > DS, indicating that soils in DY and DZ might have produced more negative effects on microorganisms.

### Diversity and Compositions of Bacterial Community

The high-throughput sequencing is a powerful and precise tool for exploring diversity of complex bacterial communities. The popularity of this method is ever growing (Guo et al., 2017; He et al., 2017). In the present study, the high-throughput sequencing was used to evaluate the diversity and abundance of bacterial communities in soils contaminated with heavy metals as a result of mining. The OTU numbers showed a slightly change among ditches with different levels of pollution. Similarly, to our results, Li X. et al. (2017) reported similar OTU numbers of microbial communities in long-term moderate and severe heavy-metal contamination. However, the diversity of soil bacteria (Shannon) in the studied soils showed significant change. Some researchers have concluded that high concentrations of heavy metals exert a significant impact on soil bacterial community and reduce the values of Shannon’s indexes (Jose et al., 2011). However, a growing metal stress in soils may result in changes on microbial diversity, which relies on the initial conditions of the system and can either decrease or increase (Giller et al., 2009). In our research, the highest bacterial diversity was found in DY. Soils from this site was severely contaminated with heavy metals. It is possible that the highest bacterial diversity in severed-contaminated areas was a result of development of the acquired tolerance after adaptation to metal stress, which in turn can lead to an increase in bacterial diversity. Adaption in the bacterial community was reported for metal-contaminated soils, where bacterial richness remained high in long-term Cr-, Zn-, and Hg- polluted soil compared to unpolluted soils (Bourceret et al., 2016; Frossard et al., 2018).

Proteobacteria, the predominant phyla in all nine soil samples, was insensitive to heavy metal pollution. Multiple reports suggest that Proteobacteria constitute the main phylum in environments with high concentrations of heavy metal, such as uranium mine soil (Sar et al., 2007), arsenic mine sediments (Halte et al., 2011), heavy metal-polluted river, sediments, soils (Serkebaeva et al., 2013; Yin et al., 2015; Li X. et al., 2017) and electronic waste region with heavy metal pollution (Wu et al., 2017). The strong adaptability of Proteobacteria to heavy metals can be possibly explained by their complex lifestyles and use of the various energy sources (Bouskill et al., 2010). Janssen (2006) studied the microbial community in unpolluted soil which was different from those revealed in our soils in which *Verrucomicrobia* and *Planctomycetes* were dominant in normal soil but were not found in our research. Long term heavy metal pollution have resulted in a remarkable changes in bacterial communities structure of soils examined in current research compared to those of previous studied unpolluted soils (Janssen, 2006). Changes in the bacterial community may be attributed to the already occurred selection of stress tolerant groups of microorganisms, while, at the same time, number of sensitive to stress bacterial species was reduced.

Further taxonomic classification at the genus level revealed more detailed information on the bacterial community structure. *Thiobacillus, Sphingomonas*, and *Nitrospira* were prevailing genera in all soil samples. Species from these genera are known to be predominant in metal-contaminated environments and can resist heavy metal toxicity (Li X. F. et al., 2015; Nitzsche et al., 2015; Guo et al., 2017). It was reported that *Thiobacillus* species were often found in bioleaching systems that contain various heavy metals (Fowler and Crundwell, 1998; Li X. et al., 2017). Other studies have demonstrated that *Nitrospira* and *Sphingomonas* can degrade a variety of toxic organic compounds (Baraniecki et al., 2002; Song et al., 2015) which could co-exist with heavy metals at contaminated sites. Considerably, the dominance of particular groups of microorganisms might be attributed to their tolerance to high concentrations of heavy metals.

In addition, several genera were predominant in DY and DZ, including *Arthrobacter, Streptomyces, Bradyrhizobium*, and *Rhizomicrobium*. According to previous reports, species in these genera exhibited various responses to heavy metals (Akobe et al., 2007; Rastogi et al., 2010). For instance, the exopolysaccharides in *Arthrobacter* sp. carry the ability to remove Cr (VI) without adjusting pH (Li Y. M. et al., 2015). Nies (1999) found that *Streptomyces* could precipitate and agglomerate colloidal suspensions in water by producing the flocculating active metabolite and releasing it to the extracellular environment. This metabolite can mitigate the toxicity of heavy metals. The ability of *Bradyrhizobium* to absorb metal ions has been reported (Cao et al., 2017). On the other hand, Guo et al. (2017) reported that species of *Rhizomicrobium* are heavy metal sensitive, since their abundance in soil negatively correlate with content of heavy metals. Some microorganisms that are tolerant to heavy metal can slowly and constantly repair the quality of soil by reducing the valences of heavy metal ions or changing contents of heavy metals, and therefore heavy-metal-sensitive microorganisms can survive in the presence of such microbes. However, this hypothesis about microbial ‘cooperation’ still requires conformation. *Pseudomonas* was the most predominant genus in DZ3 and is known as an extremophilic bacterium with strong adaptability. It is one of the most diversified bacterial genera broadly used in the field of environmental pollution control and biotechnology (Lustman et al., 2015; Yin et al., 2016; Feng et al., 2018; Singh et al., 2019). Some recent studies have reported that the *Pseudomonas* exert impact on the toxicity of heavy metals by redox (Rastogi et al., 2010; Cao et al., 2017). Moreover, Choudhary and Sar (2011) demonstrated that *Pseudomonas* could accumulate uranium within the cell envelope while maintaining its viability, indicating that the predominance abundance of *Pseudomonas* in DZ3 might be related to higher uranium concentration.

Overall, heavy metal tolerance involved different mechanisms including, biosorption, bioaccumulation, precipitation by extracellular polymeric substances and redox transformations. These dominant genera which could tolerate heavy metal toxicity and grow in contaminated soil have the potential for bioremediation of the heavy metal contaminated soil.

### Relationships Between Environmental Parameters and Bacterial Community Structures

Several researchers suggested that uranium had a great influence on soil bacterial communities. Rastogi et al. (2010) found that high toxic metal levels exerted high pressure on indigenous microbial communities in uranium mine. Sar et al. (2007) revealed that microbial communities were shifted after long-term uranium pollution and around 20% of clone sequences may represent new organisms adapted this pollution environment. In this study, uranium contamination was the most serious in the studied area, but uranium had little effect on bacterial communities compared with Cr and As. In natural environment, uranium mainly exists in U (VI) which is less toxic to bacteria. Moreover, Lixiviation experiments conducted by Mondani et al. (2011) indicate that 0.1% of uranium was water soluble in nature uranium-rich soils, which provided further evidence that the content of uranium to which bacteria are really exposed. The soil was highly polluted with uranium (95.37 mg kg^–1^) in the sampling site, though the non-residual fraction was only 1.9%. Therefore, the low impact may be attributed to the low bioavailable uranium content. Furthermore, microbes can potentially affect uranium migration by various processes as well. Microorganisms that inhabited in radionuclide contaminated soil were well-adapted to this harsh conditions (Satchanska et al., 2004; Pollmann et al., 2005), since they played an important role in the mobility of U through making altered chemical complexes (Merroun et al., 2006; Beazley et al., 2007) and redox reactions (Beller, 2005).

Some authors revealed that heavy metal pollution did not exert a significant impact on soil bacterial community (Roy et al., 2004; Zhang et al., 2013). In this study, SOM and pH were the dominant factors influencing bacterial communities. Soil organic matter was of vital importance for the ecological function of soil. It stimulated microbial growth, since it is the main source of energy and nutrients for soil microbiota (Ondrasek et al., 2019). The significant relationships between SOM and microorganisms could be observed. Moreover, SOM participated in the making chemical complexes and chelation of heavy metals, which could affect the migration and transformation of heavy metals. Kirkham (2006) suggested that SOM was one of the most important factors influencing the bioavailability of heavy metals in soil. Study performed by Chodak et al. (2013) concluded that the effect of heavy metals on soil microorganisms was weaker compared with the effects of soil pH. Soil pH was known to affect mobility and thus bioavailability of heavy metals, since it could transform inactive forms of heavy metals into active forms (McBride et al., 1997; Zhang et al., 2017). The weaker influence of heavy metals on microbial communities compared with these environmental factors might due to the mitigation of the toxic effect of metal by environment (Nannipieri et al., 2011), or by the low-level of metal biological availability (Wang et al., 2007a). Specifically, Cr was the key metal related to the regulation of the bacterial community in the studied area. Our results are in line with Li B. X. et al. (2017) who reported that Cr content was the major metal affecting soil bacterial community. Cr is widely distributed in some soil environments as a result of its extensive use in various industrial production, such as electrofacing, corrosion control, and leather-tanning processes. Unlike other heavy metals, Cr exert discrepant effects in different chemical forms and therefore it is deemed to be a prime example among other elements. The trivalent chromite [Cr (III)] and hexavalent chromate [Cr (VI)] are the most stable valence states of Cr in various forms and therefore they are predominant in the natural environment (Elzinga and Cirmo, 2010). The compounds of Cr (III) are extremely stable in soil since it entirely precipitates. In contrast, Cr (VI) is highly unstable and remain immobilized in soils (Kabata-Pendias, 2010). Therefore, microbial-induced conversion of Cr (VI) to Cr (III) leads to a reduction in toxicity, which may be a result of the precipitation of various Cr (III) chemical forms. Moreover, a toxicity has been observed even in soils containing very low contents of Cr (VI) (<1 mg kg^–1^) (Martí et al., 2013). Min et al. (2017) reported that total Cr content affiliated with Cr (VI) was the major factor for the difference of soil bacterial communities. Similar results can be found in works of He et al. (2016). The findings of these studies supported the different impact of Cr, indicating that the influence of Cr is more dependent on its occurrence state. In our cases, the percentage of non-residual Cr was relatively high, suggesting that more Cr were bioavailable in studied area, and the results compared well with those of previous studies.

Based on 16S rRNA high throughput sequencing, we provided a detailed insight into bacterial communities associated with uranium mine. Bacterial community displayed higher diversity in DY and DZ than those in DS, while bacterial richness was not significant different among three ditches. SOM, Cr and pH were the key factors that had the great impact on microbiota, other were less important. More correlations between non-residual Cr and dominant bacterial abundance were observed. Among dominant genera, the abundances of *Sphingomonas* and *Pseudomonas* were significant and positively correlated with the concentrations of non-residual Cr, U, and As. Compared with total content of heavy metal, soil physicochemical properties and non-residual heavy metal could impact a more important role in understanding bacterial communities interaction with environments.

## Data Availability

The raw reads had been submitted to NCBI and the accession numbers of sequences for DY1, DY2, DY3, DZ1, DZ2, DZ3, DS1, DS2 and DS3 are SRR9611297, SRR9611293, SRR9611298, SRR9611292, SRR9611295, SRR9603206, SRR9611294, SRR9611299, and SRR9611296.

## Author Contributions

QZ collected the samples from uranium mine. SX and QZ was responsible for conceiving the idea of this study. SX carried out the analyses reported here, involved in the writing and finalizing of the manuscript and all presented data. XC supervised and directed the entire project and obtained funds for carrying out these studies. HC detected the chemical elements. ML, FD, and IA revised the manuscript. All authors read and approved the final manuscript.

## Conflict of Interest Statement

The authors declare that the research was conducted in the absence of any commercial or financial relationships that could be construed as a potential conflict of interest.
