# Supplementary material for: Speciation Distribution of Heavy Metals in Uranium Mining Impacted Soils and Impact on Bacterial Community Revealed by High-Throughput Sequencing
Source: Front Microbiol. 2019 Aug 13;10:1867. doi: 10.3389/fmicb.2019.01867 (PMC6700481; doi:10.3389/fmicb.2019.01867)
Supplement: Supplementary file 1 [file Data_Sheet_1.docx]

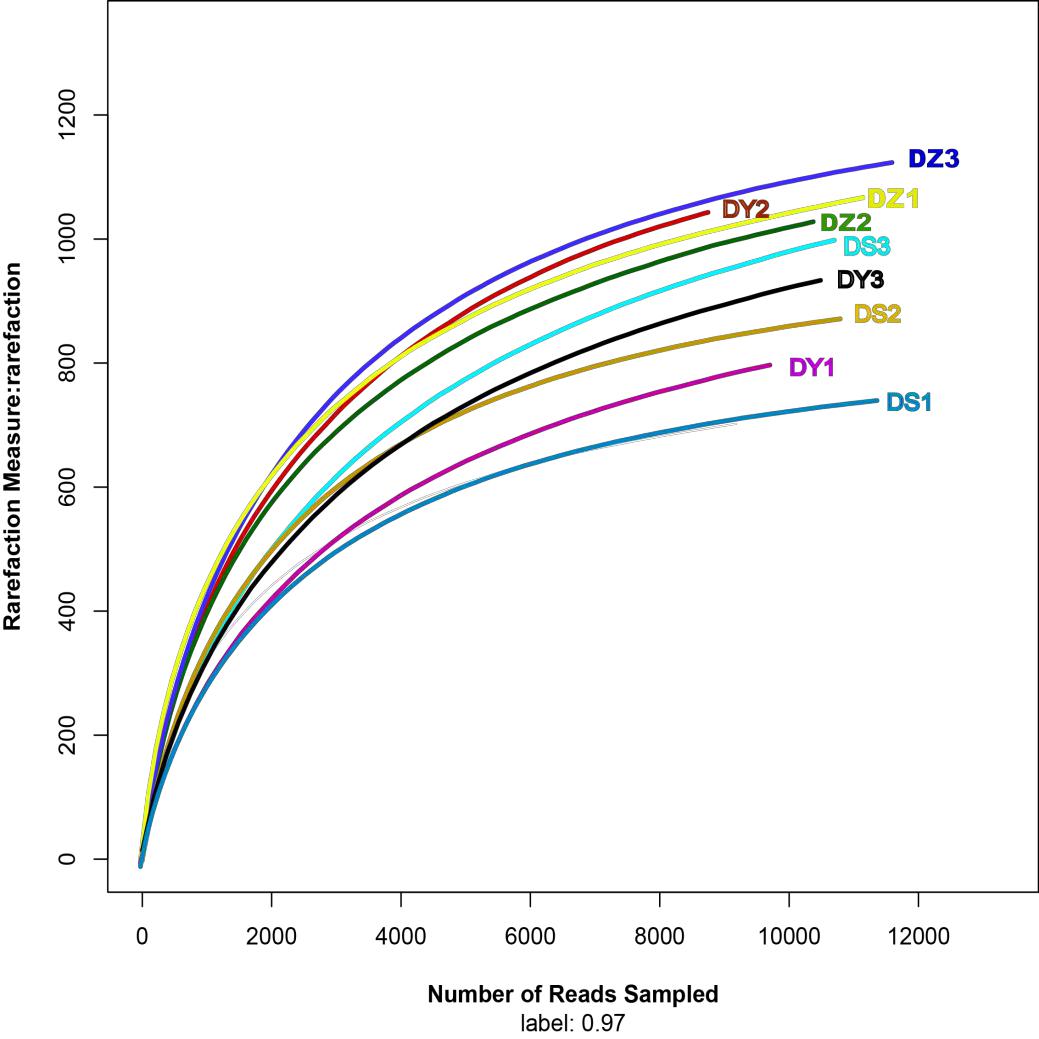


1

1. **Fig. S1.** Rarefaction curves show the number of unique OTUs (sharing≥97% sequence identity)
2. per total reads of nine samples. OTU counts as a function of sequence depth.
